# Outdoor Exercise for People with Rheumatic Disease–Study Protocol and Baseline Characteristics of the OUTdoor Physical ACtivity Cohort (OUTPAC)

**DOI:** 10.3390/ijerph22010092

**Published:** 2025-01-11

**Authors:** Jonas R. Ahler, Lars H. Tang, Dorthe V. Poulsen, Søren T. Skou, Pætur M. Holm

**Affiliations:** 1 The Research and Implementation Unit PROgrez, Department of Physiotherapy and Occupational Therapy, Næstved-Slagelse-Ringsted Hospitals, Region Zealand, 4200 Slagelse, Denmark; larta@regionsjaelland.dk (L.H.T.); stskou@health.sdu.dk (S.T.S.); pamh@regionsjaelland.dk (P.M.H.); 2 Department of Regional Health Research, University of Southern Denmark, 5000 Odense, Denmark; 3 Department of Geosciences and Natural Resource Management, University of Copenhagen, Rolighedsvej 23, 1958 Frederiksberg C, Denmark; dvp@ign.ku.dk; 4 Center for Muscle and Joint Health, Department of Sports Science and Clinical Biomechanics, University of Southern Denmark, 5230 Odense, Denmark; 5 Faculty of Health Sciences, University of Faroe Islands, 100 Tórshavn, Faroe Islands; 6 Center for Surgery, National Hospital of Faroe Islands, 100 Tórshavn, Faroe Islands

**Keywords:** nature, outdoor, physical activity, rheumatic disease, cohort study, intervention

## Abstract

The OUTPAC cohort study evaluates the setup and implementation of a nationwide Danish initiative focused on the impact of structured outdoor physical activity (PA) on individuals with rheumatic diseases. This prospective cohort study includes more than 1600 participants, predominantly women (92%), with an average age of 65 years (range: 28–93). The cohort primarily consists of individuals with osteoarthritis (72%), rheumatoid arthritis (18%) and nonspecific lower back pain (13%). Volunteer instructors conducted interventions in outdoor settings, targeting strength, balance, physical capacity, mental health, and interaction with nature. Data collection involved questionnaires and physical tests in four primary outcome domains: quality of life, pain, physical function and activity, and mental health. Despite moderate pain (VAS mean: 48.3), high medication use (71%), and serious fatigue (54%), participants indicated having a good quality of life (EQ-5D-5L mean: 0.81) and average mental health (WHO-5 mean: 62.9). Baseline scores from physical tests showed results comparable to the general population aged 60–69 years. The OUTPAC project offers clinical insight into the implications of outdoor PA interventions on individuals with rheumatic disease while shedding light on the development and implementation of a large-scale nationwide outdoor PA intervention. Future analyses will examine short- and long-term changes and potential determinants.

## 1. Introduction

Rheumatic diseases such as osteoarthritis (OA) and rheumatoid arthritis (RA) are among the primary causes of disability worldwide [1,2]. Engaging in and maintaining a physically active lifestyle is a cornerstone recommendation for the management of rheumatic disease and is associated with improved physical and mental health [3,4,5]. However, individuals living with rheumatic disease often struggle with sustained motivation and adherence to clinical recommendations for physical activity (PA) [6,7]. To motivate more people with rheumatic disease to be more physically active, it is important to offer engaging, accessible, and social environments that address common barriers for participation such as time constraints and lack of motivation [8,9]. In recent years, the outdoor environment as a setting for therapeutic efforts has gained attention. Research highlights its positive impact on mental health, including a reduction in stress, depression, and anxiety [10,11]. Structured outdoor PA programs tailored to a specific disease may improve health-related quality of life (HRQOL), physical function, and mental well-being [12]. Moreover, studies suggest that outdoor PA enhances health perception, increases energy levels and encourages sustained participation more effectively than indoor exercise [13,14,15,16,17]. However, this is based on a few studies of low overall quality and only two studies have specifically focused on people with rheumatic disease, highlighting the need for more research on this population [12].

In 2022, the Danish Rheumatism Association, in collaboration with the Danish Gymnastic and Sport Organization (DGI) and the research and implementation unit PROgrez at Næstved-Slagelse-Ringsted hospitals, launched a nationwide Danish initiative for outdoor exercise. Embedded within this nationwide initiative was OUTdoor Physical ACtivity (OUTPAC), a prospective cohort study evaluating the short- and long-term effects of outdoor PA on individuals with rheumatic disease. Additionally, the project aimed to identify potential predictors of change in individuals’ physical function and activity, HRQOL, mental outcome and pain, and, using socio-demographics, the medical, physical and mental variables of people with rheumatic disease. In this paper, we provide an overview of the setup and implementation of the cohort as well as baseline characteristics of the participants included in the OUTPAC project.

## 2. Methods

### 2.1. Study Design

This paper is reported according to STrengthening the Reporting of OBservational studies in Epidemiology (STROBE) guidelines [18] and follows ethical regulations and the Declaration of Helsinki. Region Zealand’s ethics committee on health research waived approval (EMN-2021-09728) and approval was obtained from the Danish Data Protection Agency (REG-147-2021).

### 2.2. Nature Settings

The outdoor intervention took place nationwide across Denmark with great variety in the outdoor setting, but mainly took place in woods, urban parks, beaches and local outdoor parking lots. Nature interactions were used intentionally [19], as trees, branches, rocks, and leaves were used as exercise tools and invited the participants to engage in a more playful way of exercising. Sensory experience was enhanced by listening, smelling and touching the outdoor surroundings, particularly in exercises with a mental focus. Furthermore, the aesthetically pleasing surroundings were expected to be a motivational factor as well as stimulate social engagement [20,21].

### 2.3. Participants

The outdoor intervention was offered by the Danish Rheumatism Association, primarily aimed at individuals with rheumatic diseases such as OA or RA. Despite this focus, there were no specific exclusion criteria, allowing participants without rheumatic disease as well. Since the program was advertised through the Danish Rheumatism Association, most participants would have some form of rheumatic disease.

### 2.4. Study Enrollment

Participants were enrolled by responding (either online or by phone) to advertisements on social and local media. In addition, hospitals, general practitioners, and primary care physiotherapists were informed about the initiative and encouraged to share the information with people with rheumatic disease. Upon registration for the outdoor intervention, participants paid a registration fee of 150 DKK (€ 20). As part of enrollment, participants received information about the OUTPAC study and provided written consent to participate.

### 2.5. Instructor Training

There were no specific professional requirements for volunteers to sign up as an instructor. This meant that anyone, health professional or not, with or without prior experience as an instructor, could sign up as a volunteer instructor. All instructors had to take part in a mandatory two-day training course led by physiotherapists experienced in structured outdoor PA. The instructors also received training in the outdoor exercises and applying physical performance-based tests (see Section 2.7.1). The instructors had sole responsibility for the outdoor intervention and were required to include both physical and mental exercises (see the examples in Table 1) and incorporate the outdoor environment into the exercises. The instructors were provided with a guidebook with information about exercise in general, mental health, pain, how to be an instructor in an outdoor setting, and various suggestions for exercises. The pain section provided guidance for instructors on managing participants’ pain during exercises and addressing how pain may change, for better or worse, following exercise. The guidebook provided two examples of a ‘master class’. Each master class contained 10 to 15 predefined outdoor exercises that lasted 60 min in total which the instructors could choose to follow.

### 2.6. Characteristics of the Outdoor Intervention

Based on previous experience with outdoor exercise aimed at people with anxiety, stress or depression [22], the DGI and the Danish Rheumatism Association modified the outdoor intervention to fit people with rheumatic disease. The Research and Implementation Unit PROgrez was tasked with evaluating the outdoor intervention, which involved selecting appropriate physical tests and developing questionnaires.

The exercises were not standardized but varied in focus and intensity; some focused on strength, balance and physical capacity while other exercises focused predominantly on the mental aspect and interaction with the outdoor setting (Table 1). With a focus on movement, community, engagement and having a good experience, the intervention aimed to stimulate the heart rate, balance, senses, and enjoyment.

Each exercise session was performed face-to-face in groups of a maximum of 12 participants, had a duration of 60 min and was performed once a week for 12 weeks. The first groups receiving the intervention started in the spring of 2022, with new teams starting in the fall of that year. This was repeated in 2023, with teams initiating the intervention both in the spring and fall of that year. The outdoor interventions were available in 50 different locations spread across Denmark, covering all five Danish regions (see Supplementary S1).

### 2.7. Data Collection

**Figure 1:**
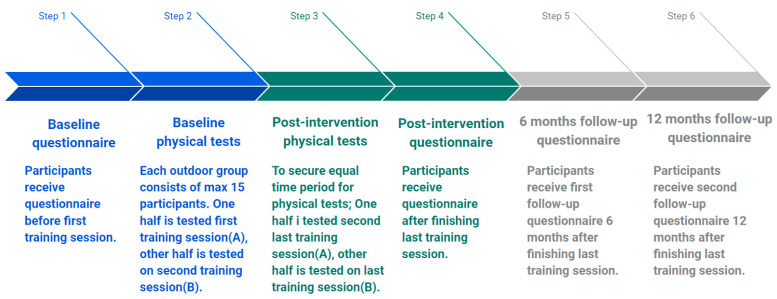
Timepoints for measurements.

Participants consented to undergo two physical tests, both before and after the intervention period, and to complete four online questionnaires: before the intervention; after the 12-week intervention; 6 months after the last training session; and 12 months after the last training session (see Figure 1). Any non-responders to online questionnaires received automatic reminders after one and two weeks.

All participants enrolled in the OUTPAC study were registered in the REDCap database [23], along with their questionnaire and physical performance-based test data, using a distinct ID and their email address.

#### 2.7.1. Physical Tests

The physical performance-based tests (30-s chair stand [24] and 40-m walking test [24]) were performed in the natural environment and adapted accordingly (e.g., using tree stumps or park benches). The instructor’s guidebook included a table to enter participants’ ID number, age and test results. For logistical reasons, the instructors were not able to test all 12 participants in one training session. Therefore, half of the participants were tested during the first and second-to-last training sessions while the other half were tested during the second and last training sessions, i.e., the test results reflected 11 weeks instead of the full 12-week intervention.

#### 2.7.2. Outcome Measures

The OUTPAC project predetermined four key outcome domains, which were quality of life, pain, physical function and activity, and mental outcomes. Each outcome domain consisted of validated questionnaires supplemented by single-item questions. The Oswestry Disability Index (ODI) was only made available to participants if they answered the lower or upper back as their most troubled body region. The questionnaires also included questions about the participants’ medical history, exercise habits and overall expectations of the outdoor PA intervention. The following is an overview of the key outcome domains:

- *Health-related quality-of-life:* EuroQol-5 Domain (EQ-5D) and EuroQol visual analogue scale (EQ VAS).
- *Physical function and activity:* Oswestry Disability Index (ODI), UCLA activity score, and the mean scores from two physical performance-based tests, 40-m walking test and 30-s chair test.
- *Pain:* The visual analogue scale (VAS) was included in the questionnaires. Pain-related questions about the number of affected joints and use of pain medication were included in the pain analyses.
- *Mental outcomes:* The World Health Organization-Five Well-Being Index (WHO-5) was included in the questionnaire. In addition, questions related to mental aspects like fatigue were included in the mental outcomes analyses.

### 2.8. Patient and Public Involvement

A reference group consisting of people living with rheumatic disease contributed to the development, understandability and adaption of the questionnaires. This was supplemented by further input and adaptation by the Danish Rheumatism Association and the DGI.

## 3. Results

In the OUTPAC project, 50 different locations were used with one instructor at each location. A total of 1943 participants were enrolled between 21 February 2022 and 14 August 2023. The first 237 participants were enrolled in the spring of 2022, followed by an additional 488 in the fall of 2022. Subsequently, 772 participants were enrolled in the spring of 2023, and 446 participants in the fall of 2023.

Of the 1943 participants receiving the baseline questionnaire, 1648 responders had complete outcome data (85%) while 107 had incomplete outcome data (5%). The remaining 188 registered participants (10%) were non-responders due to withdrawal of consent, not wanting to answer the questionnaire or other unknown reasons. An overview of the baseline characteristics for participants in the OUTPAC project is presented in Table 2 while an expanded version of Table 2 is available in Supplementary S2.

### 3.1. Participants and Clinical Characteristics

The participants were, on average, 66 years old. Most were retired, 1017 (60%), while 286 (17%) had an ordinary full-time job and 180 (11%) had part-time jobs. Moreover, 1576 (92%) of the participants were women. In the cohort, 1262 (72%) had OA, 310 (18%) had RA, and 224 (13%) had nonspecific lower back pain. Besides participants’ rheumatic disease, 1299 (75%) had additional diseases (see full list in Supplementary S3). The three most common troubled joints were the knee, 821 (46%), wrists/fingers, 800 (45%) and lower back, 796 (45%). The joints attributed to most daily difficulties were the knee, 419 (25%), lower back, 320 (19%) and wrists/fingers, 256 (15%). A total of 932 (54%) participants reported trouble walking due to arthritis or muscle and joint pain. A total of 499 (29%) participants reported at least one fall in the past 12 months and 1058 (83%) experienced some degree of fatigue, with 681 (54%) experiencing moderate, severe or very severe fatigue.

### 3.2. Questionnaires and Physical Tests

In the quality-of-life domain, the mean score of the EQ-5D-5L scale was 0.81 and the EQ-VAS had a mean of 61.9. The mean score of the VAS was 48.3, the UCLA activity score showed a mean of 5.27 and the ODI mean score was 25.2%. In the mental outcome domain, the mean score of the WHO-5 was 62.9. On the physical tests, 748 (38%) completed the 30-s chair test and averaged 14 repetitions while 754 (39%) completed the 40-m walking test with an average completion time of 27.9 s, meaning an average walking speed of 1.4 m/s. An overview of the results is presented in Table 3.

## 4. Discussion

This study offers new insights into people with rheumatic disease who enroll in outdoor interventions. The OUTPAC project, involving more than 1600 participants, consisted mainly of women with an average age of 66 years. The baseline data of the cohort showed OA, RA, and nonspecific lower back pain as the most common conditions with a large presence of comorbidity, moderate pain, frequent use of pain medication and serious fatigue. Additionally, the results from the EQ-5D-5L and EQ-VAS show a good quality of life and the results of the WHO-5 consider this cohort to fall within the average of the general population [25]. Based on the results of the physical performance-based tests, the average physical function of the cohort is in the vicinity of the general population aged 60–69 years [24].

### 4.1. Characteristics of the Population

The OUTPAC cohort’s characteristics are consistent with the evidence of people with rheumatic disease: OA, RA, and back disorders are the most prevalent characteristics [1], with an average age of 65.5 years [26], and 75% reported having a disease other than rheumatic disease [27,28,29]. Notably, most of the participants were women (92%). This distribution of women is higher than the 70% reported in the large Danish osteoarthritis registry, Good Life with Osteoarthritis in Denmark (GLA:D^®^) [30], comprising more than 70,000 participants. Despite a higher OA prevalence among women globally (approx. 60% [31]) and a higher distribution of women members of the Danish Rheumatism Association (74%) [unpublished data], the nine-out-of-ten (92%) distribution of women comes as a surprise. Evidence suggests men are less likely to maintain healthy behaviors, including having difficulty engaging in healthy practices, and in health promotion interventions [32,33]. This could be one reason that explains the low number of men enrolled in OUTPAC. Nonetheless, this gender imbalance could affect the generalizability of the results.

The cohort includes 69% of participants who exercise at least once a week and 40% who do so twice weekly (see Supplementary S2). This indicates that many participants are already active. Therefore, for some, the outdoor intervention will supplement existing activity, while for others, it will introduce primary PA. However, GLA:D^®^-registry data suggests that pain reduction is independent of baseline activity level, meaning participants with different activity habits may benefit similarly [34].

### 4.2. Health-Related Quality-of-Life, Physical Function and Activity, Pain, and Mental Outcomes

Participants in the cohort reported a relatively high quality of life, though their scores were below the general population average, which is 0.92 on the EQ-5D-5L and 86.0 on the EQ-VAS [35,36]. The cohort’s scores were 0.81 on the EQ-5D-5L scale and 61.9 on the EQ-VAS and the difference in scores aligns with existing evidence indicating rheumatic disease negatively affects quality of life [37]. Given the literature indicates outdoor PA can improve health perception and the EQ-5D-5L assesses mobility, self care, activity, pain and anxiety or depression, an outdoor intervention could potentially enhance quality of life [12,13,14,15,16,17]. However, since rheumatic disease may affect these domains differently, longitudinal investigations of OUTPAC will aim to distinguish between different types of rheumatic disease and their impact on the quality-of-life domain.

In relation to pain, participants reported an average pain intensity of 48.3, predominantly affecting the knees, lower back, and wrists/fingers, which is consistent with established evidence on joint pain location and intensity [37,38]. Notably, 71% of participants reported having used pain medication in the past 2 weeks. Although extensive use of pain medication corresponds with the evidence [39], regular use of pain medication can be damaging and long-term use of pain medication specifically for OA is problematic and generally advised against [40,41]. Previous evidence shows PA can reduce pain in people with rheumatic disease [42], however, the impact of outdoor interventions on pain, as well as their influence on different types of rheumatic disease, has been scarcely investigated [12].

Mental outcomes at baseline showed a mean score of 62.9 on the WHO-5, indicative of an average mental well-being comparable to the general population [25]. Across various types of chronic disease, participation in structured outdoor interventions seems to improve mental health outcomes [12]. Yet, considering the average score lies within the general population’s range, there seems to be only small room for improvement. Nonetheless, evidence suggests nature can significantly reduce mental symptoms like stress by reducing cortisol levels [43,44], making further investigation on the influence of outdoor interventions on stress, particularly in individuals with lower mental health scores, and of potential variations of intervention between types of rheumatic disease relevant. Furthermore, more than half of the participants reported moderate to extreme fatigue over the past week, a common symptom in various forms of arthritis that can severely impact quality of life by reducing PA levels [45]. As such, outdoor PA can improve energy [13,14,15,16,17] and the influence of an outdoor intervention on energy level, including fatigue, would be highly relevant for further investigation.

For the physical function and activity domain, participants completed an average of 14 repetitions in the 30-s chair test, which is a bit higher than the baseline results from the GLA:D^®^-registry, which stand at 12 repetitions [30]. These results align with normative data for community-dwelling older adults aged 65–69 years, where women typically achieve 11 to 16 repetitions and men 12 to 18 [24]. However, in GLA:D^®^, the participants improved from 12 to 15 repetitions after a 6–8-week exercise program twice a week [30]. Future analyses will determine whether similar changes can be obtained from outdoor interventions. In the 40-m walking test, the average walking speed was 1.4 m/s, close to the GLA:D^®^-registry baseline of 1.5 m/s, yet slower than the reference values of 1.8 m/s for women and 1.9 m/s for men aged 60 to 69 years, which represent healthy individuals [24,30]. In GLA:D^®^, improvements were made from 1.5 m/s to 1.6 m/s [30], which would indicate a small but clinically relevant difference [46]. Later analyses will determine whether similar changes can be gained from an outdoor intervention and any potential variation between different types of rheumatic disease.

### 4.3. Limitations and Considerations

The OUTPAC study was shaped by the comprehensive outdoor intervention framework established by the Danish Rheumatism Association, predetermining key elements such as participant eligibility, intervention design, and instructor requirements, drawing on previous experience to inform these decisions.

The lack of participant eligibility criteria is a potential issue. As shown in Table 2, 14% of the participants do not have a known rheumatic disease, which complicates the generalizability of the results for people with rheumatic disease. The instructors play an important role and influence participant adherence and maintenance of an intervention [47]. As the instructors were volunteers, the requirements had to be lowered to recruit and educate enough to manage a large request for intervention. This could lead to varying levels of exercise quality, experience in managing a group, adaptability to participants’ needs and overall knowledge of rheumatic diseases. These factors could affect intervention delivery. In contrast, broad enrolment criteria allowed the intervention to be offered in 50 different locations, enabling more than 1600 people to participate, which would not have been possible with more strict inclusion criteria.

The absence of predetermined specific exercises and the time allocated to each exercise invariably creates a broad range of exercise program variations. Some instructors may focus primarily on physical exercises, while others favor mental exercises, and as a result, interventions may differ significantly across instructors and locations. Conversely, a less rigid exercise program may enable instructors to better tailor exercises to the needs of both the group and individuals, though a lack of experience on the instructor’s part could present challenges. Importantly, since the Danish Rheumatism Association refers to the intervention as ‘nature training’, distinguishing between indirect, incidental and intentional interactions with nature is necessary, as the level of interaction could influence the outcome [19]. A scoping review [48] highlights how natural environments, with varied terrain and elements, can improve balance and prevent falls in older adults by encouraging diverse movement that enhances strength and balance. This knowledge can be used to create more engaging, nature-based exercise alternatives to traditional machine-based routines.

In the OUTPAC study, the surroundings vary between woods, urban areas, beaches and parking lots. The impact of the intervention on participants’ mental health could be diminished if the natural surroundings lacked certain elements, such as biodiversity, the presence of water, and various types of trees, bushes, and flowers [49]. Additionally, exercising in an urban environment with people and cars passing by may be perceived as stressful [50]. Furthermore, detailed reporting of the diverse natural settings across the 50 locations could have provided valuable insights.

The amount of missing data from the physical tests, with less than half of registered participants having a baseline score for both tests, was due to a combination of decision regret after signing up and before partaking in the intervention and inability to complete the intervention, along with other unknown study attrition reasons. A small number of instructors failed to submit baseline test results and did not respond to multiple contact attempts. Lastly, the data collection did not capture information on adverse events, or any negative experiences associated with participation in the exercise intervention.

## 5. Conclusions

This baseline report from the OUTPAC project offers valuable clinical insight into more than 1600 participants with rheumatic diseases, while also shedding light on the development and implementation of a large-scale nationwide outdoor PA intervention. Despite challenges in collecting physical test data, the high questionnaire response rate offers a robust foundation for analysis. The baseline cohort data reveal that OA, RA and nonspecific lower back pain are prevalent, often accompanied by comorbidity, moderate pain, frequent use of pain medication and fatigue. Future research will assess the impact of structured outdoor PA on pain, physical activity and function, mental health and HRQOL in individuals with rheumatic disease, as well as compare outcomes across different conditions.

## Figures and Tables

**Table 1:** Examples of exercises from the instructor’s guidebook.

Exercise	Focus
**Obstacle course**	An obstacle course through the woods with sticks, tree stumps and stones. Create two teams and complete the obstacle course with different variations like speed, without vision or backwards.
**Walk in nature**	Walk through nature by forming a line behind the instructor. With a two-meter distance between participants, the task is to walk in silence through nature and experience the environment without talking.
**Breathing** **exercises and** **visualizations**	Stand with equal weight distribution and experience your breathing, preferably with your eyes closed. Smell, feel and listen to your surroundings. Visualize different experiences in your body, such as feeling hot/cold, tense/relaxed, pain/no pain.
**Stationary** **training**	Do a series of different exercises which challenge your strength, balance and physical capacity. This could be to run/walk on the same spot, walk along a fallen tree or lift a heavy stone.

**Table 2:** The baseline characteristics of participants in the OUTPAC project.

Category		Mean (SD) or n (%)
Participant Characteristics		
Age (years)	All individuals (n = 1494)	65.5 (8.2)
BMI (kg/m^2^)	All individuals (n = 1677)	27.2 (5.0)
Gender (n = 1717)	Woman	1576 (91.8)
	Man	137 (8.0)
	Other	4 (0.2)
Educational level (n = 1687)	No education or primary school	103 (6.1)
	Secondary education	51 (3.0)
	Vocational education	377 (22.4)
	Short-term education (2–3 years)	299 (17.7)
	Middle-term education (3–4 years)	693 (41.1)
	Long-term education (>4 years)	164 (9.7)
Occupational status (n = 1684)	Ordinary work	286 (17.0)
	Part-time work	180 (10.7)
	On sick leave	66 (3.9)
	Unemployed	14 (0.8)
	Student	5 (0.3)
	Retirement	1017 (60.4)
	Private support	21 (1.3)
	Other	95 (5.6)
**Clinical Characteristics**		
Rheumatic or pain condition (Select multiple if relevant) (n = 1735)	Osteoarthritis	1254 (72.3)
	Rheumatoid arthritis	308 (17.8)
	Non-specific lower back pain	224 (12.9)
	Osteoporosis	194 (11.2)
	Herniated disc	188 (10.8)
	Spinal stenosis	124 (7.2)
	Fibromyalgia	119 (6.9)
	Vertebral collapse	73 (4.2)
	Psoriatic arthritis	65 (3.8)
	Polymyalgia/temporal arteritis	48 (2.8)
	Sjögren’s syndrome	35 (2.0)
	Spondyloarthritis (Bechterew’s disease)	34 (2.0)
	Gout	32 (1.8)
	Lupus (systemic lupus erythematosus)	18 (1.0)
	Other rheumatic disease	60 (3.5)
	Muscle or joint pain without known rheumatic disease	224 (12.9)
	No muscle or joint pain or known rheumatic disease	22 (1.3)
Comorbidity (Full list available in Supplementary S3) (n = 1724)	No additional disease besides arthritis	425 (24.7)
	One comorbidity	521 (30.2)
	Two comorbidities	405 (23.5)
	More than two comorbidities	373 (21.6)
Pain medication in the past 2 weeks (n = 1709)	Used pain medication in the past 2 weeks	1213 (71.0)
	Did not use pain medication in the past 2 weeks	496 (29.0)
Type of pain medication (Select multiple if relevant) (n = 1208)	Paracetamol	1103 (91.3)
	NSAID, NSAID as cream or acetylsalicylic acid	589 (48.8)
	Corticosteroid injection	46 (3.8)
	Opioid (morphine, tramadol, codeine)	147 (12.2)
	Antidepressant (for neuropathic pain)	63 (5.2)
	Anticonvulsant	51 (4.2)
	Medical cannabis	11 (0.9)
	Other	121 (10.0)
Previous surgery (n = 1736)	Previous surgery in a joint	743 (42.8)
	No previous surgery in a joint	993 (57.2)
Joint pain during the last 24 h (Select multiple if relevant) (n = 1709)	Foot/ankle	608 (34.3)
	Knee	821 (46.3)
	Hip	547 (30.8)
	Lower back	796 (44.9)
	Upper back (from mid-back and above)	267 (15.1)
	Neck	520 (29.3)
	Shoulder	558 (31.5)
	Elbow	121 (6.8)
	Wrist/fingers	800 (45.1)
	Other	49 (2.8)
	Not experienced pain in the last 24 h	98 (5.5)
Most troubled joint (n = 1708)	Foot/ankle	190 (11.1)
	Knee	419 (24.5)
	Hip	184 (10.8)
	Lower back	320 (18.7)
	Upper back (from mid-back and above)	62 (3.6)
	Neck	113 (6.6)
	Shoulder	110 (6.5)
	Elbow	8 (0.5)
	Wrist/fingers	256 (15.0)
	Other	26 (1.5)
	Not experienced difficulty with any joints	20 (1.2)
Debut of pain or functional impairment (n = 1712)	No history of pain or functional impairment	34 (2.0)
	0–12 months ago	109 (5.8)
	1–3 years ago	240 (14.1)
	4–10 years ago	453 (26.6)
	+10 years ago	876 (51.5)
Walking (n = 1735)	Walking difficulty due to condition	932 (53.7)
	No walking difficulty	803 (46.3)
Fall in the last 12 months (n = 1730)	No fall	1231 (71.2)
	One or two falls	438 (25.3)
	Three or more falls	61 (3.5)
Fatigue * (n = 1272)	No fatigue	214 (16.8)
	Mild	377 (29.6)
	Moderate	470 (37.0)
	Severe	189 (14.9)
	Very severe	22 (1.7)

* The fatigue-related question was added to the questionnaire later in the project.

**Table 3:** Results in key outcome domains with validated outcome measurements.

Outcome Domain	Validated Questionnaires and Physical Tests	Number of Participants	Mean (SD)
Quality of life	EuroQol-5 Domain index (EQ-5D-5L)	1697	0.81 (0.17)
	EuroQol visual analogue scale (EQ VAS)	1693	61.9 (19.9)
Pain	Visual analogue scale (VAS)	1693	48.3 (23.2)
Physical actvity and function	Oswestry Disability Index (ODI) *	379	25.2% (12.3)
	UCLA activity score	1708	5.27 (1.36)
	EQ-5D-5L (mobility)	1688	0.97 (0.03)
	40-m walking test	748	27.9 s (7.8)
	30-s chair test	754	14 repetitions (4.4)
Mental outcomes	The World Health Organisation-Five Well-Being Index (WHO-5)	1695	62.9 (18.8)

* Only available to participants with lower/upper back as their most troubled joint.

## Data Availability

The authors do not have permission to share data.

## Supplementary Materials

The following supporting information can be downloaded at: https://www.mdpi.com/article/10.3390/ijerph22010092/s1, Supplementary S1: Locations in Denmark, Supplementary S2: Expanded Table 2, Supplementary S3: Full list of other diseases.
