# Supplementary material for: Outdoor Exercise for People with Rheumatic Disease–Study Protocol and Baseline Characteristics of the OUTdoor Physical ACtivity Cohort (OUTPAC)
Source: Int J Environ Res Public Health. 2025 Jan 11;22(1):92. doi: 10.3390/ijerph22010092 (PMC11765233; doi:10.3390/ijerph22010092)
Supplement: Supplementary file 1 [file ijerph-22-00092-s001.zip › ijerph-3394967-supplementary.pdf]

Supplementary materials for article:

"Outdoor exercise for people with rheumatic diseases - baseline characteristics of the OUTdoor Physical ACtivity cohort (OUTPAC)"

Supplemental materials: **2**

Supplementary S1: Locations in Denmark for structured outdoor PA interventions in spring 2023. 2

Supplementary S2: Expanded table 2: 3

Supplementary S3: Full list of other diseases than arthritis with results: 8

Supplemental materials:

Supplementary S1: Locations in Denmark for structured outdoor PA interventions in spring 2023.

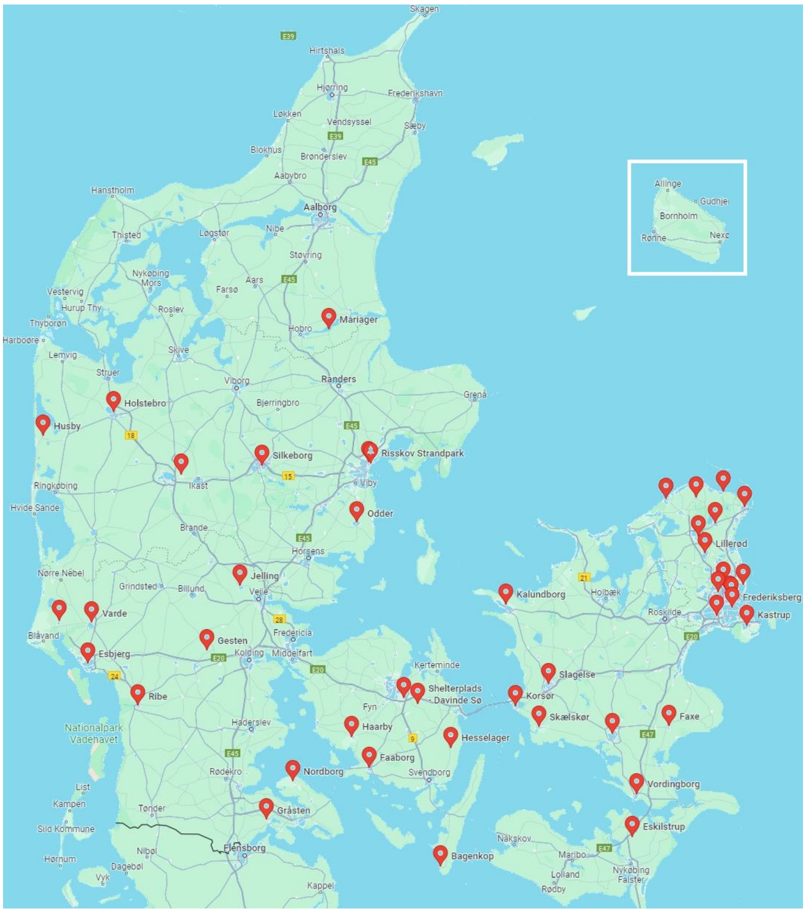

Supplementary S2: Expanded table 2:

Category	Question from baseline questionnaire	Response options	Number of responders	Mean (SD)
**Participant characteristics**				
Age (years)	State your age	 All individuals<br>Men<br>Women<br>Other	 1.494<br>110<br>1.334<br>3	65.5<br>(8.2)<br>69 (7.8)<br>65.1<br>(8.2)<br>67 (1.0)
BMI (kg/m2)<br>(mean and SD)	State your weight in kilo<br>State your height in centimeter<br>(Calculated from height and weight)	All individuals<br>Men<br>Women<br>Other	1.677<br>133<br>1.539<br>4	27.2<br>(5.0)<br>28.7<br>(5.1)<br>27.1<br>(5.0)<br>26.1<br>(0.7)
				**Percent age**
Sex	State your gender	Women<br>Men<br>Other	1.576<br>137<br>4	91.8%<br>8.0%<br>0.2%
Educational level	What is the highest education level you have completed?	No education or primary school<br>Secondary education<br>Vocational education<br>Short-term education (2-3 years)<br>Middle-term education (3-4 years)<br>Long-term education (> 4 years)	103<br>51<br>377<br>299<br>693<br>164	6.1%<br>3%<br>22.4%<br>17.7%<br>41.1%<br>9.7%
Occupational status	What is your current occupational status?	Ordinary work or part-time work less than 30<br>hours weekly<br>Flexjob<br>On sick leave<br>Unemployed<br>Student<br>Senior pension / Early retirement pension / State<br>pension / Early retirement scheme<br>Private support<br>Other	 350<br>116<br>66<br>14<br>5<br>1017<br>21<br>95	 20.8%<br>6.9%<br>3.9%<br>0.8%<br>0.3%<br>60.4%<br>1.3%<br>5.6%
**Clinical characteristics**				
Arthritis or pain condition	What type(s) of arthritis or muscle	Osteoarthritis<br>Rheumatoid arthritis<br>Gout	1,254<br>308<br>32	72.3%<br>17.8%<br>1.8%

	and joint pain do you have? (Select multiple if relevant)	Polymyalgia/Temporal arteritis	48	2.8%
		Psoriatic arthritis	65	3.8%
		Osteoporosis	194	11.2%
		Fibromyalgia	119	6.9%
		Lupus (Systemic lupus erythematosus)	18	1,0%
		Spondyloarthritis (Bechterew's disease)	34	2.0%
		Sjögren's syndrome	35	2,0%
		Herniated disc	188	10.8%
		Spinal stenosis	124	7.2%
		Vertebral collapse	73	4.2%
		Non-specific lower back pain (Lower back pain without known cause)	224	12.9%
		Other rheumatic diseases	60	3.5%
		I have pain in muscles or joints, but do not have a rheumatic disease	224	12.9%
		I have neither a rheumatic disease nor pain in muscles or joints	22	1.3%
Comorbidities (Full list available in supplementary 3)	Besides your arthritis, do you have any other disorders/illnesses? (Select multiple if relevant)	No additional diseases besides arthritis	425	24.7%
		1 comorbidity	521	30.2%
		2 comorbidities	405	23.5%
		More than 2 comorbidities	373	21.6%
Pain medication	Have you taken pain-relieving medication in the past 2 weeks? (excluding herbal medicine/dietary supplements)	Yes I have	1.213	71%
		No I have not	496	29%
Types of pain medication	What painkillers have you taken in the last 2 weeks? (Select multiple if relevant)	Paracetamol (e.g., Pamol, Panodil, Pinex)		
		NSAIDs and/or acetylsalicylic acid (e.g.,		
		Kodimagnyl, Ibumetin, Diclofenac, Bonyl)	1.103	91.3%
		NSAIDs as cream (topical treatment - e.g.,	417	34.5%
		Voltaren cream)	172	14.2%
		Corticosteroid injection (e.g., cortisone)	46	3.8%
		Morphine (e.g., Methadone, Oxynorm, Ketogan,	51	4.2%
		morphine patch)	61	5.0%
		Tramadol (e.g., Dolol, Gemadol, Tradolan)	35	2.9%
		Codeine (e.g., Kodipar)	63	5.2%
		Antidepressants (for neuropathic pain)	51	4.2%
		Anticonvulsants (e.g., Gabapentin,	11	0.9%
		Carbamazepine, Tegretol)	121	10.0%
		Medical cannabis		
		Other		
Alternative pain medication	Have you taken any other forms of therapeutic arthritis medication? (Select multiple if relevant)	Methotrexate (e.g., Emthexate)	137	7.7%
		Bisphosphonate	4	0.2%
		Biologic medication	105	5.9%
		Other medication	102	5.8%
		No, I have not	923	52.0%

Previous operation	Have you previously undergone surgery on a joint in your body?<br>(e.g., knee, hip, or back)	Yes, I have previously been operated in a joint<br>No, I have not previously been operated in a joint	743<br>993	42.8%<br>57.2%
Previous operated joints	Which joint(s) have you previously had surgery on?<br>(Select multiple if relevant)	Foot/ankle<br>Knee<br>Hip<br>Lower back<br>Upper back (from the middle part of the back and upwards)<br>Neck<br>Shoulder<br>Elbow<br>Wrist/fingers<br>Other	138<br>385<br>132<br>121<br>12<br>44<br>139<br>26<br>161<br>25	7.8%<br>21.7%<br>7.4%<br>6.8%<br>0.7%<br>2.5%<br>7.8%<br>1.5%<br>9.1%<br>1.4%
Walking	Do you have walking difficulties as a result of your arthritis or muscle and joint pain?	Yes, I have trouble walking caused by my condition<br>No, I do not have trouble walking caused by my condition	932<br>803	53.7%<br>46.3%
Fall in the last 12 months	Within the last year, have you experienced a fall?<br>(A fall is defined as an unintentional event that resulted in you ending up lying/sitting on the ground, floor, or another lower level—either with or without loss of consciousness or injury)	No<br>Yes, 1 time<br>Yes, 2 times<br>Yes, 3 or more times	1.231<br>306<br>132<br>61	71.2%<br>17.7%<br>7.6%<br>3.5%
Debut for pain or function	When did you first experience a decrease in function or pain in muscle or joints?	I have not experienced a decrease in function or pain in muscle or joints<br>0 - 6 months ago<br>6 - 12 months ago<br>1 - 3 years ago<br>4 - 10 years ago<br>+ 10 years ago	34<br>40<br>59<br>240<br>453<br>876	2.0%<br>2.3%<br>3.5%<br>14.1%<br>26.6%<br>51.5%
Specify joint pain	Specify which joint(s) you have experienced pain in within the last 24 hours.	Foot/ankle<br>Knee<br>Hip<br>Lower back<br>Upper back (from mid-back and above)<br>Neck<br>Shoulder<br>Elbow	608<br>821<br>547<br>796<br>267<br>520<br>558<br>121	34.3%<br>46.3%<br>30.8%<br>44.9%<br>15.1%<br>29.3%<br>31.5%<br>6.8%

		Wrist/fingers	800	45.1%
		Other	49	2.8%
		I have not experienced pain in the last 24 hours	98	5.5%
Most troubled joint	Which joint have you overall experienced the most difficulty with?	Foot/ankle	190	11.1%
		Knee	419	24.5%
		Hip	184	10.8%
		Lower back	320	18.7%
		Upper back (from mid-back and above)	62	3.6%
		Neck	113	6.6%
		Shoulder	110	6.5%
		Elbow	8	0.5%
		Wrist/fingers	256	15.0%
		Other	26	1.5%
		I have not experienced difficulty with any joints.	20	1.2%
Fatigue	This question is about the specific type of fatigue characterized by energy depletion, exhaustion, or tiredness that doesn't improve with rest or sleep.<br>In the last 7 days, how would you generally rate your fatigue?	No fatigue	214	16.8%
		Mild	377	29.6%
		Moderate	470	37.0%
		Severe	189	14.9%
		Very severe	22	1.7%
**Additional Questions**				
Information on physical activity	Before the OUTPAC Project, have you received information about the importance of physical activity and exercise?<br>(e.g., from a doctor, nurse, physiotherapist, or similar)	Yes, I have received information about the impact of physical activity	1.377	79.5%
		No, I have not received information about the impact of physical activity	356	20.5%
Received information about weight loss	Before the OUTPAC Project, have you received information about losing weight?<br>(e.g., from a doctor, nurse, dietitian, or similar)	Yes, I have received information about the impact of weight loss	406	23.4%
		No, I have not received information about the impact of weight loss	1.330	76.6%
Level of physical activity	Compared to others your age, do you consider yourself?	Much less physically active	150	8.8%
		Somewhat less physically active	508	29.9%
		About the same level of physical activity	657	38.7%
		Somewhat more physically active	330	19.5%

		Much more physically active	53	3.1%
Physical activity frequency	How often do you usually exercise to the point of being out of breath or sweating?	Never<br>Less than once a month<br>1-2 times a month<br>Once a week<br>2-3 times a week<br>4-6 times a week<br>Every day	170<br>201<br>165<br>486<br>538<br>92<br>47	10.0%<br>11.8%<br>9.7%<br>28.6%<br>31.7%<br>5.4%<br>2.8%
Physical activity duration	On average, how long do you exercise per session when/if you do exercise?	Less than 15 minutes<br>15-30 minutes<br>31-60 minutes<br>More than 1 hour	249<br>449<br>831<br>168	14.7%<br>26.4%<br>49.0%<br>9.9%
Physical activity effort	How much effort do you typically exert when you exercise?	I exercise gently<br>I get out of breath and sweat<br>I push myself to near exhaustion	979<br>691<br>28	57.7%<br>40.7%<br>1.6%
Afraid of physical activity	Are you afraid that your joints might be harmed by physical activity and exercise?"	Yes I am<br>No I am not	216<br>1.475	12.8%<br>87.2%
Symptom management	Arthritis symptoms and pain can vary in intensity. How do you typically manage temporary worsening of your arthritis symptoms and pain? (Select multiple if relevant)	I remain completely still and avoid all activities<br>that cause pain<br>I adjust my activity level according to my pain<br>I continue to use my joint as I normally would<br>I take pain medication/more pain medication<br>Relaxation exercises, Mindfulness or mental<br>training<br>Acupuncture<br>Heat<br>Cold<br>Massage<br>Other	 92<br>1.276<br>278<br>849<br>394<br>70<br>404<br>119<br>305<br>138	 5.2%<br>71.9%<br>15.7%<br>47.9%<br>22.2%<br>3.9%<br>22.8%<br>6.7%<br>17.2%<br>7.8%
Source of information	Where did you hear about the opportunity to participate in outdoor training?	Personal doctor<br>Personal network (friends, family, colleagues)<br>Local media<br>Social media (Facebook, Arthritis Association's<br>website, etc.)<br>Through the hospital (e.g., during outpatient<br>appointments or hospitalization)<br>From municipal/clinic physiotherapist<br>Other	 4<br>196<br>136<br>1.234<br>5<br>5<br>104	 0.2%<br>11.6%<br>8.1%<br>73.3%<br>0.3%<br>0.3%<br>6.2%
Overall expectations	What overall expectations do you have for improving your arthritis after participating in the OUTPAC Project?	Very significant or complete improvement<br>Significant improvement<br>Moderate improvement<br>Slight improvement<br>Very slight or no improvement	18<br>304<br>917<br>342<br>103	1.1%<br>18.1%<br>54.4%<br>20.3%<br>6.1%

Expect to continue	Do you expect to continue with outdoor training after the project ends?	Yes, definitely	269	16.0%
		Yes, possibly	1.015	60.3%
		Don't know	381	22.6%
		No, doubtful	16	0.9%
		No, not at all	3	0.2%

Supplementary S3: Full list of other diseases than arthritis with results:

**Question from baseline questionnaire**	**Response options**	**Number of responders**	**Percentage**
Besides your arthritis, do you have any other disorders/illnesses? (Select multiple if relevant)	Elevated blood pressure (hypertension)	596	33.6%
	Elevated cholesterol	477	26.9%
	Chronic heart failure	9	0.5%
	Ischemic heart disease (e.g., angina (chest pain due to heart problems), heart attack, bypass surgery, or balloon angioplasty)	32	1.8%
	Other heart disease	79	4.5%
	Poor circulation in the legs (e.g., peripheral artery disease)	94	5.3%
	Anemia (reduced amount of red blood cells/anemia) or other blood disorder	13	0.7%
	Stroke (including consequences after stroke)	41	2.3%
	Parkinson's disease	1	0.1%
	Dementia	0	0.0%
	Other neurological disease (e.g., multiple sclerosis, epilepsy, or migraine)	79	4.5%
	Diabetes type 1 (diabetes, where the body completely stops producing	8	0.5%
	insulin)	51	2.9%
	Diabetes type 2 (diabetes, where the body cannot use insulin as well as it		
	should/not producing enough insulin)	178	10.0%
	Metabolic disorder (high or low metabolism)	17	1.0%
	Kidney disease	121	6.8%
	Stomach ulcer or other stomach disease (e.g., gastritis or reflux)	160	9.0%
	Bowel disease (e.g., irritable bowel syndrome or Crohn's disease)	13	0.7%
	Liver disease	37	2.1%
	Chronic obstructive pulmonary disease (COPD)	155	8.7%
	Asthma	70	4.0%
	Other lung and respiratory disease	83	4.7%
	Cancer - within the last 5 years (excluding minor cases of skin cancer)	96	5.4%
	Psoriasis	100	5.6%
	Depression	81	4.6%
	Anxiety	150	8.5%
	Other chronic illness	425	24.0%
	No diseases other than arthritis		
